# Psychosocial Correlates of Dual Methods for Contraception and STI Protection in Urban Adolescents

**DOI:** 10.5402/2011/469610

**Published:** 2011-10-29

**Authors:** Robert P. Pack, Xiaoming Li, Bonita F. Stanton, Lesley A. Cottrell

**Affiliations:** ^1^Department of Community Health, College of Public Health, East Tennessee State University, P.O. Box 70623, Johnson City, TN 37614, USA; ^2^Prevention Research Center, Department of Pediatrics, Wayne State University School of Medicine, Detroit, MI 48201, USA; ^3^Department of Pediatrics, West Virginia University School of Medicine, Morgantown, WV 26506, USA

## Abstract

*Purpose*. To identify correlates of combined hormonal contraception and condom use (dual method use) compared with no methods, condoms only or hormonal contraception only. Data are from a baseline assessment of 335 youth (52% female) enrolled in an intervention trial. Multinomial logistic regression identified theory-based factors associated with dual method use. At last intercourse 47% of respondents used dual methods, 29% condom only, 14% hormonal contraception only, and 10% no methods. *No method* users were less likely than *dual-method* users to feel “dirty” about pregnancy, to have ask about historical condom use, to have more than two partners, to view condom use as normative for boys and more likely to perceive pregnancy risk as remote. *Hormonal-contraception-only* users were more likely to have sex weekly and perceive sex as pleasurable for girls, and less likely to view condom use as normative for boys and to ask a partner to use a condom. *Condom-only* users were more likely to perceive pregnancy chance as remote, and less likely to have more than two partners and to want peers to think they were virgins. Interventions should include benefits of dual methods while counseling about the negative impact of STI and unplanned teen pregnancy.

## 1. Introduction

Aside from abstinence, consistent condom use is the best protection against sexually transmitted diseases (STD), including human immunodeficiency virus (HIV). Hormonal contraception methods such as birth control pills, the intrauterine device (IUD), Norplant^®^, and Depo-Provera^®^ are clearly the best method for protection against pregnancy. Some adolescents use dual methods for protection against both pregnancy and STD, a desirable behavior that should encouraged. However, those who do use either or both methods are more likely to do so for pregnancy prevention than to prevent STD or HIV [1].

### 1.1. Prevalence

Several studies have examined dual-method use, and the prevalence of dual use varies slightly by method and sample; ranging from 14 to 21%. Crosby and colleagues found that, among their sample of young women entering into a study at STD clinics, about 14% reported dual-method-use over time [2]. Santelli and colleagues found that about 17% of their community-based sample of teenage and adult women used dual methods [3]. When surveying a nationally representative sample of males, Lindberg and colleagues found that about 17% used a condom when they thought or knew that their female partner was using a hormonal method [4]. In a follow-up study of 104 of 13–25-year-old British women who had received family planning clinic services, and who were initially prescribed the birth control pill, 23 (22%) reported using both hormonal contraception and condoms [5]. 

Consistent dual-method use is yet another matter. In a study of six separate administrations of a Youth Risk Behavior Survey, with nationally representative samples ranging from 10,904 to 16,262 youth, Anderson and colleagues found that consistent dual use increased significantly over the decade from 1991 to 2001 (from 3.6%–7.2%) but remained rather low. Those who were older and who were not minorities had the highest dual-use prevalence [6]. 

Prevalence of dual-method use at last intercourse is considerably higher. In a cross-national study of 15-year-old young women, in 24 countries, between 14.1% (Croatia) and 37.6% (England) of respondents reported sexual activity. Among those, dual use at last intercourse ranged from 2.6% in Croatia to 28.8% in Canada [7]. Pazol and colleagues studied contraceptive practices of 15–44-year-old women. They found that, at last intercourse, 45% of women aged 15–19-years-old, 24.1% of women aged 20–24, and 9.1% of women older than 25 reported dual-method use. Dual-method use was also related to having multiple partners in this sample [8]. 

In the U.S., Healthy People 2020 goals exist for dual-method-use for youth. Specifically, for dual-method use at last intercourse, the goal is that, before the year 2020, 20.2% of 15–19-year-old women will use dual methods, against a baseline of 18.4% in 2008 [9]. 

### 1.2. Correlates

Correlates and predictors of dual-method use can assist in the development of intervention programming to increase use. Santelli and colleagues examined correlates of dual contraceptive use in a nationally representative sample of youth [10]. They found that condom use at last intercourse was reported by a quarter of young men whose partner was using the pill. Correlates of dual use among men included younger age, black race, engaging in fewer nonsexual risk behaviors, and having received instruction about HIV in school. Among young women, about a fifth of those who used the pill for birth control reported also using a condom at last intercourse. Correlates of dual-method use for females included: younger age, black race and fewer nonsexual risk behaviors, having no partners in the previous three months, older age at first sex, and having talked to parents or other adult relatives about HIV [10].

Crosby examined correlates of any female hormonal method used in combination with condoms. Correlates of dual use among this sample of young women included a strong desire to avoid pregnancy, parents knowing whom they were with, and higher levels of parent communication [2]. 

Similarly, Seiving and colleagues found that adolescent's perceptions of parental expectations about sex and contraception, and their attitudes about practical social and moral implications of birth control were all significant correlates of dual use. Moreover, they found that dual-method use at most recent intercourse was as high as 25% in one wave of their study [11]. 

Santelli and colleagues, in their community-based study of young women, also found that positive attitudes toward safer sex, ever having refused sex without a condom, and believing in condom efficacy were all statistically significant correlates of dual-method use for young women; however, having ever been tested for HIV was negatively related to dual-method use [3]. 

In a cross-sectional study of 453 Hispanic and African-American adolescents who were recently (past four weeks) sexually active, and who came to a reproductive health clinic, Roye [12] found that adolescents who used oral contraceptives or long-acting hormonal methods (i.e., Depo-Provera or Norplant) were less likely to have used a condom in the last four weeks than teens whose only method of birth control was condoms. Only those teens who had previously been diagnosed with a STD were more likely to have used a condom [12].

Stanton and colleagues [13] conducted a longitudinal study of 383 African American youth aged 9 to 15 years enrolled in a randomized, controlled trial of an HIV risk reduction intervention. They found that about two thirds of those who used oral contraceptives also used condoms in the past. Having received an AIDS education intervention and having higher level of knowledge about HIV/AIDS was associated with use of more effective contraceptive practices (e.g., prescription or nonprescription methods of birth control in addition to condoms). Posttest analysis revealed that more than 80% of the youth who used oral contraceptives also used condoms. Moreover, contraceptive practices showed considerable stability over time [13]. 

In a sample of older subjects, aged 18–29, de Visser found that dual use was not related to concerns about HIV/STD or unplanned pregnancy. Rather, he found that dual use was correlated with partner type, communication, and positive attitudes and intentions toward condom use [14].

At least one clinic-based intervention study has shown significant promise in promoting dual use. Peipert and colleagues reported on a provider-based, staging-theory-driven intervention among an STI clinic population. They found that intervention participants had a 70% greater likelihood of dual use after 24 months, when compared to control condition participants [15].

### 1.3. Summary

From the available literature, we conclude that education and knowledge about unplanned pregnancy and STI, positive perceptions of protective behavior, positive attitudes about protective behavior, and communication are all consistent predictors of dual-method use. All of these correlates are consistent with social cognitive theoretical frameworks. The guiding theoretical model for this work is one such model: Protection Motivation Theory (PMT). In this model, risk protection motivation intention and action is a function of two main pathways: the threat- and coping-appraisal pathways. The threat appraisal pathway considers the intrinsic and extrinsic rewards minus perceived threat and vulnerability. The coping-appraisal pathway considers the perceived self- and response-efficacy of protective action minus the response cost of doing so. The theory is fundamentally a social cognitive theory, in that the person's behavior, their cognition, and the environment are all interlinked in reciprocal action [16].

### 1.4. Purpose

The purpose of the present study is to describe the prevalence of-, and theory-based psychosocial correlates of dual-method use. Our ultimate aim is to improve the understanding of theory-based predictors of dual use in order to design elements of intervention programs aimed at increasing dual use. Based on previous studies, we hypothesize that higher knowledge and negative attitudes and perceptions of sex, pregnancy, and STI will be correlated with dual contraceptive use. Specifically we examine these correlates in light of those who, at last sex, did not use condoms or other birth control (None), hormonal birth control-only users (BC only), condom-only users (CU only), those who used both methods at once (Dual).

## 2. Methods

Recruitment sites were selected to identify youth living in public housing developments in and around Baltimore, Maryland. Housing development tenants and recreational center staff were invited to assist in identifying eligible youth in these settings and in explaining the program to the youth and their parents. Ultimately 817 youth from 32 housing developments were recruited from 1999 to 2000. Among these, 358 reported having had prior sexual experience at baseline; among these youth, 335 provided data regarding contraceptive use. For more details, see Stanton, 2004 [17]. The baseline assessment data are used in the current study. The research protocol was approved by the Institutional Review Board at the University of Maryland. Written informed consent/assent was obtained from both parents and youth.

### 2.1. Measures

Participants completed a youth risk assessment questionnaire (The Youth Health Risk Behavior Inventory, YHRBI) [18] in their homes. The questionnaires were administered aurally and visually by a talking Macintosh computer and required approximately 45 minutes to complete. The YHRBI assessed youth demographic characteristics (e.g., school ranking, church attendance), sexual history (e.g., had sex in previous 6 months, had sex at least weekly, live with partner), and sexual risk behaviors (e.g., had two or more sexual partners, had STD last 6 months). In addition, the YHRBI also contained questions about youth perceptions regarding sex and condom use (e.g., intrinsic reward, social influence, intention) and AIDS knowledge. 

Use of contraception during last sexual episode was assessed by self-report. Youth were asked about what they or their partner used last time they had sex among a list of nine forms of contraception: birth control pills, condom, withdrawal; Diaphragm, Foam/jelly; sponge, Norplant, Depo-Provera, and other. The response choice for each contraception include “yes”, “no”, and “do not know”. Based on their response to the contraception question, four groups of adolescents were identified: those who did not use condoms or other birth control (None), hormonal birth control-only users (BC only), condom-only users (CU only), those who use both methods at once (Dual). 

### 2.2. Statistical Analysis

The Cochran Mantel-Haenzel test was used to identify significant differences among the four groups for each of the proposed correlates. Using the group of statistically significant associations (*P* <  .05) from the first stage of the analysis, multinomial logistic regression was used to identify the correlates of each type of contraceptive use in turn (i.e., None, Condom Use-only [CU], or hormonal birth control-only [BC]) compared to dual use [Dual], the reference group. Multinomial logistic regression is similar to conventional logistic regression but is used when the dependent variable has more than two categories. Again, since there were four distinct groups with one of them serving as the reference group, this process yielded three distinct comparisons: None versus Dual, BC versus Dual, and CU versus Dual. 

## 3. Results

### 3.1. Demographics and Behavior

Demographic and behavioral characteristics are found in Table 1. Only sexually active adolescents were included in the study. Mean age was approximately 14.5 years and about half of the subjects (52%) were female. At last sex, ten percent of the sample reported using no birth control or condoms [None], 14% reported hormonal birth control use only [BC], 29% reported condom use only [CU], and 47% reported using hormonal birth control methods and condom use together [Dual]. 

There were no significant differences in the percentage of each group that is female, the average age between groups, or the proportion in each group that identifies themselves as one of the best students in school, attending church regularly, having gotten pregnant (or gotten someone pregnant) in the last six months, or having had a child. 

There were differences between the four groups in the proportion of participants who had sex in the last six months, had sex at least weekly, had more than two sex partners, lived with their sex partner and had an STI in the last six months. In each case listed above, except living with their sex partner, the hormonal birth control-only users [BC] reported the behavior or condition with the greatest frequency. 

### 3.2. Knowledge Attitudes and Perceptions

Knowledge, attitudes, and perceptions of sex, pregnancy, and STD were compared between the four methods. The results of this analysis are found in Table 2. 

Categories of predictors included items that reflected constructs that are linked to motivation to engage in protective behaviors such as attitudes toward sex, self-reported intrinsic rewards for sex, social and peer influences, expectations about future behavior (intentions), attitudes toward condom use, and AIDS knowledge. Each category of predictor had several theory-based questions. 

Statistically significant differences in the respondent's attitudes toward sex included: feeling like they want kids their age to think they are a virgin (Dual = highest), feeling “dirty” about pregnancy (Dual = highest), and feeling “bad” about having sex (None = highest). Importantly, though the “Dual, BC, CU; or None” use category for which the correlate was most frequently endorsed is noted here, the actual meaningful differences and statistically significant results, may be between any of the four categories (e.g., Dual versus None rather than Dual versus BC).

Statistically significant differences in the respondent's self-reported intrinsic rewards for sex included: reporting that sex feels good for girls (BC = highest) and reporting that their peers use condoms (CU = highest). 

Statistically significant differences in the social and peer influences that respondents reported included the belief that most boys use condoms (CU and Dual = highest) and friends respect you less if you are pregnant (Dual = highest).

Differences in expectations about their behavior in the future included feeling more likely to get pregnant in the next six months (None = highest), feeling that it is likely that they will have sex in the next six months (BC = highest), feeling likely that they will use a condom in the next six months (CU = highest).

Statistically significant differences in attitudes toward condom use included adolescents reporting that they could get a condom if needed (all were high but BC = highest), having “asked about the number of people my partner had been with” (Dual = highest), and having asked partner about condom use (Dual = highest).

Statistically significant differences in AIDS knowledge were found between the groups. The value was indicated by the correct responses to the following: AIDS is caused by a virus (BC = highest), AIDS can be acquired with one sexual contact (BC = highest), only receptive anal sex transmits AIDS (BC = highest).

### 3.3. Models of Dual Use Compared to Each Other Behavior

The models for dual-method use were based on the theoretically plausible bivariate relationships identified above. The results are found in Table 3.

In the first model those respondents who reported using no contraception were significantly more likely than dual-method users to endorse the statement “no boys I know use condoms” (OR = 1.67; *P* < .05), to feel proud about being involved in a pregnancy (OR = 1.79; *P* < .05), and to report feeling that it is likely that they will be pregnant in the next 6 months (OR = 1.68; *P* < .05). They were also less likely to have had more than 2 sex partners (OR = .58; *P* < .05) or to have asked a partner to use a condom (OR = .14; *P* < .05). 

In the second model those who reported using birth control-only were more likely than dual-method users to endorse the statement that sex feels good for girls (OR = 1.81; *P* < .05), to endorse the statement that “no boys I know use condoms” (OR = 1.56; *P* < .05), and to have sex weekly (OR = 1.63; *P* < .05). They were less likely than dual users to have asked a partner to have used a condom (OR = .28; *P* < .05). 

In the third model, those respondents who reported using condoms only were more likely than dual method users to report feeling likely that they would get pregnant or get someone pregnant in the next six months (OR = 1.47; *P* < .05). They were less likely to have had more than two partners (OR = .75; *P* < .05), and less likely to report wanting kids their age to think they were a virgin (OR = .70; *P* < .05).

## 4. Discussion

In this study, adolescents who employed dual methods endorsed different attitudinal motivators that may influence their final decision with regard to the type and level of protection. Our findings are consistent with the theoretical framework that protection motivation is linked with perceptions of rewards and costs and that the interplay between motivators is associated with one's final protective decisions [16]. For example, adolescents who used two methods at once were different in terms of their feelings and perceptions about pregnancy, especially negative feelings about the pregnancy outcome, and their perception of their own risk for pregnancy (which was low). Based on these findings, the dual-method adolescent group appears to be more concerned about the outcome of unprotected sex, particularly a pregnancy, than other adolescents. Dual-method adolescents, therefore, appear to take more precaution to avoid those outcomes. Future work needs to examine why they are more inclined to worry about pregnancy than the others; for example, is this group naturally precautious, or do they believe themselves to have more to lose from an unplanned pregnancy than other adolescents? In one instance our findings are counterintuitive: condom-only users, as compared to dual users, were less likely to report having more than two sex partners in their life. One might expect that dual users may even try to minimize risk by minimizing number of different partners, but our data do not support this.

In contrast, adolescents who use BC only had very high levels of knowledge about HIV/AIDS and had significantly higher levels of sexual frequency than dual-method users but were more likely to consider intrinsic rewards of having sex without a condom (e.g., sex feels good for girls and boys). Additionally, adolescents in the BC-only group were also more likely to endorse peer sexual involvement. Bivariate relationships revealed heavy peer influences for CU-only behavior though these are not reflected in the multinomial regression model.

Clearly, the perspective of youth who engaged in each of the individual categories of contraceptive behavior is different from that of dual users, who are more likely to perceive greater risk and negative expectations about risky sex outcomes (pregnancy and STI). The literature confirms that dual use is more common for young women who are using hormonal contraception and who seek to protect themselves from STI risk [19].

This study has several limitations. First, it is cross-sectional and does not allow for a test of our model over time. Second, it relies on self-reported perceptions and behavior for both the independent and dependent variables; there is no assurance that those who reported dual methods do so every time they had sex. In fact, the extant literature does not support high rates of consistent dual use [6]. In our sample, the proportion of youth in this study who reported dual use at last intercourse was about twice that of some studies that examined the same topic [2, 9] although in the same range as some recent studies [7, 8]. This is likely due to the fact that in our study we included all of the birth control methods other than condoms into one category and it is unclear if those studies included such products as contraceptive foams and jellies. 

Use of dual methods is much better for pregnancy prevention and is as good for STI prevention as CU-alone, but only if the condom is used correctly. Health educators and health care practitioners should educate sexually active youth about this distinction and encourage use of dual methods for adolescents who are not abstinent. Efforts to advocate for dual use might include discussion of the key benefits of both methods while also counseling about the negative effects of both STI and unintentional pregnancy on teen's lives. 

A focused intervention strategy might involve intensive training modules based on increasing risk perception for STI irrespective of experience with and use of birth control methods. Moreover, Peipert's stage-based approach to dual-method promotion was effective for clinic populations. Hence a staging approach alongside other PMT theory-based health education strategies may be expected to yield increases in dual-method use. Also, focused strategies can be developed if an interventionist can categorize individuals based on historical experience with different birth control methods, and carefully crafted modules can be delivered to youth that enhance risk perceptions and negative outcome expectations about risky sexual outcomes relative to that youth's (or group of youths) historical experience. 

Health educators, clinicians, and other behavioral interventionists should encourage dual use and highlight the correct and incorrect perceptions of risks relative to the many outcomes of sexual behavior. More research is needed that (1) uncovers the reasons for increased risk perception and protection motivation among dual-method users, (2) pathways to increasing that risk perception, and (3) expansion of intervention strategies and trials to adolescents who are not in traditional high-risk groups.

## Figures and Tables

**Table 1:** Demographic Characteristics.

	Contraceptive Method (last time sex)			
	None	BC	CU	Dual
*N* (%)	35 (10%)	47 (14%)	96 (29%)	157 (47%)
Female	47%	54%	43%	58%
Mean age (SD)	14.46 (1.24)	14.89 (1.11)	14.54 (1.05)	14.76 (1.04)
States they are one of the “best students”	26%	19%	24%	32%
Reports attending church weekly	29%	26%	25%	29%
Had sex in last 6 months	80%	83%	63%	75%*
Had sex at least weekly	6%	28%	8%	7%***
Had ≥2 sex partners	8%	40%	29%	29%*
Live with partner	11%	9%	0%	6%*
Had STD in the last 6 m	6%	15%	3%	6%*
Got pregnant in the last 6 m	9%	11%	4%	5%
Had children	9%	4%	3%	3%

(1) Definition of contraceptive method: None: did not used any; BC: birth control only (one of the birth control methods except condom), CU: Condom only, Dual: condom + one of other birth control methods. (2) *P* values *P < 0.05 and ***P < 0.001 were obtained by CMH Chi-square test.

**Table 2:** Perceptions of youth about sexual and protective behavior.

	Contraceptive method			
	None	BC	CU	Dual
Attitude to sex; respondent reports that:				
I want kids my age to think I am a virgin	38%	24%	23%	41%*
Kids my age would want to have sex to see how it feels	86%	75%	73%	68%
I can say no if I did not want to have sex	74%	81%	80%	88%
I feel dirty about pregnancy	15%	9%	16%	32%***
I feel bad about having sex	39%	15%	16%	27%*

Intrinsic reward; respondent reports that:				
Sex feels good for girls	41%	68%	46%	48%*
Sex feels good for boys	71%	83%	69%	63%

Social influence; respondent reports that:				
Most close friends use condom	45%	43%	65%	55%*
Most boys use condom	30%	28%	56%	52%**
Most girls have sex	58%	72%	51%	61%
Friends would respect them less if pregnant	9%	9%	9%	18%**

Intention; in the next 6 months, I am:				
Likely to get pregnant	21%	17%	10%	3%**
Likely to have sex	58%	81%	63%	58%***
Likely to use condom	64%	77%	86%	76%*

Attitude toward condom use; respondent reports that:				
I could get condom	85%	96%	88%	92%*
I have asked about the number of people my partner had sex with	57%	70%	61%	78%*
I have asked partner about use condom	49%	68%	78%	87%****
I believe that condoms take away feelings	53%	50%	32%	37%
I believe that people do not use condom in serious relationship	41%	41%	29%	30%

AIDS knowledge; respondent reports that:				
AIDS is caused by virus (yes)	85%	100%	96%	86%**
You can get AIDS in one sexual contact (yes)	85%	96%	84%	75%*
Only receptive anal sex transmits AIDS (no)	61%	85%	76%	67%*

(1) *P* value *P < 0.05, **P < 0.01, ***P < 0.001,and ****P < 0.0001 were obtained by CMH Chi-square test.

**Table 3:** Nominal regression analysis showing association of variables with each method dichotomy (final model).

	Model fit			Estimated Odds Ratios	
	*X* ^2^	*P*	None/Dual	BC/Dual	CU/Dual
Age	2.01	0.5694	0.924	1.086	0.853
Male gender	1.38	0.7093	1.131	0.946	1.156
Sex feels good for girls	8.15	0.0429	1.033	1.812**	1.027
No boys I know use condom	14.36	0.0024	1.678**	1.564**	0.952
Feels proud about pregnancy	10.81	0.0128	1.791**	1.221	1.210
Likely to get pregnant in next 6 month	10.43	0.0153	1.683*	1.306	1.474**
Had ≥ 2 sexual partners	8.49	0.0369	0.583*	0.931	0.752*
Had sex at least weekly	11.51	0.0093	0.878	1.633**	1.083
Asked partner about use condom	18.56	0.0003	0.139**	0.279**	0.618
Want kids my age to think I am virgin	9.61	0.0221	1.049	0.824	0.701**

(1) Estimated odds ratios were produced by multinomial logistic regression for the nominal dependent variable “contraceptive method”. Each model used “dual method” as the reference group. (2) The variables included in the final model were identified as significant effects on contraceptive method using Maximum Likelihood Estimation. Age and gender were included in each model as control variables. *P < 0.05, **P < 0.01.

## References

[1] Traeen B, Lewin B, Sundet JM (1992). Use of birth control pills and condoms among 17-19-year-old adolescents in Norway: contraceptive versus protective behaviour?. *AIDS Care*.

[2] Crosby RA, DiClemente RJ, Wingood GM (2001). Correlates of using dual methods for sexually transmitted diseases and pregnancy prevention among high-risk African-American female teens. *Journal of Adolescent Health*.

[3] Santelli JS, Davis M, Celentano DD, Crump AD, Burwell LG (1995). Combined use of condoms with other contraceptive methods among inner-city Baltimore women. *Family Planning Perspectives*.

[4] Lindberg LD, Ku L, Sonenstein FL (1998). Adolescent males’ combined use of condoms with partners’ use of female contraceptive methods. *Maternal and Child Health Journal*.

[5] Gregson J, Kirkman R (1999). Double Dutch: looking at the usage of combined pill plus condom in girls under 25. *European Journal of Contraception and Reproductive Health Care*.

[6] Anderson JE, Santelli J, Gilbert BC (2003). Adolescent dual use of condoms and hormonal contraception: trends and correlates 1991-2001. *Sexually Transmitted Diseases*.

[7] Godeau E, Gabhainn SN, Vignes C, Ross J, Boyce W, Todd J (2008). Contraceptive use by 15-year-old students at their last sexual intercourse: results from 24 countries. *Archives of Pediatrics and Adolescent Medicine*.

[8] Pazol K, Kramer MR, Hogue CJ (2010). Condoms for dual protection: patterns of use with highly effective contraceptive methods. *Public Health Reports*.

[9] U.S. Department of Health and Human Services Healthy people 2020: the road ahead. http://www.health.gov/healthypeople/url/.

[10] Santelli JS, Warren CW, Lowry R (1997). The use of condoms with other contraceptive methods among young men and women. *Family Planning Perspectives*.

[11] Sieving RE, Bearinger LH, Resnick MD, Pettingell S, Skay C (2007). Adolescent dual method use: relevant attitudes, normative beliefs and self-efficacy. *Journal of Adolescent Health*.

[12] Roye CF (1998). Condom use by Hispanic and African-American adolescent girls who use hormonal contraception. *Journal of Adolescent Health*.

[13] Stanton BF, Li X, Galbraith J, Feigelman S, Kaljee L (1996). Sexually transmitted diseases, human immunodeficiency virus, and pregnancy prevention: combined contraceptive practices among urban African-American early adolescents. *Archives of Pediatrics and Adolescent Medicine*.

[14] De Visser R (2007). Why do heterosexual young adults who use reliable contraception also use condoms? Results from a diary-based prospective longitudinal study. *British Journal of Health Psychology*.

[15] Peipert JF, Redding CA, Blume JD (2008). Tailored intervention to increase dual-contraceptive method use: a randomized trial to reduce unintended pregnancies and sexually transmitted infections. *American Journal of Obstetrics and Gynecology*.

[16] Rogers RW, Cacioppi T, Petty RE (1983). Cognitive and physiological processes in fear appeals and attitude change: a revised Theory of Protection Motivation. *Social Psychology*.

[17] Stanton B, Cole M, Galbraith J (2004). Randomized trial of a parent intervention: parents can make a difference in long-term adolescent risk behaviors, perceptions, and knowledge. *Archives of Pediatrics and Adolescent Medicine*.

[18] Stanton B, Li X, Black M (1995). Development of a culturally, theoretically, and developmentally based survey instrument for assessing risk behaviors among African-American early adolescents living in urban low-income neighborhoods. *AIDS Education and Prevention*.

[19] Ott MA, Adler NE, Millstein SG, Tschann JM, Ellen JM (2002). The trade-off between hormonal contraceptives and condoms among adolescents. *Perspectives on Sexual and Reproductive Health*.

